# Site-specific receptor methylation of FrzCD in *Myxococcus xanthus* is controlled by a tetra-trico peptide repeat (TPR) containing regulatory domain of the FrzF methyltransferase

**DOI:** 10.1111/j.1365-2958.2008.06323.x

**Published:** 2008-06-19

**Authors:** Ansley E Scott, Eric Simon, Samuel K Park, Philip Andrews, David R Zusman

**Affiliations:** 1 Department of Molecular and Cell Biology, University of CaliforniaBerkeley, CA, USA; 2 Department of Biological Chemistry, University of MichiganAnn Arbor, MI, USA

## Abstract

*Myxococcus xanthus* is a gliding bacterium with a complex life cycle that includes swarming, predation and fruiting body formation. Directed movements in *M. xanthus* are regulated by the Frz chemosensory system, which controls cell reversals. The Frz pathway requires the activity of FrzCD, a cytoplasmic methyl-accepting chemotaxis protein, and FrzF, a methyltransferase (CheR) containing an additional domain with three tetra trico-peptide repeats (TPRs). To investigate the role of the TPRs in FrzCD methylation, we used full-length FrzF and FrzF lacking its TPRs (FrzF^CheR^) to methylate FrzCD *in vitro*. FrzF methylated FrzCD on a single residue, E182, while FrzF^CheR^ methylated FrzCD on three residues, E168, E175 and E182, indicating that the TPRs regulate site-specific methylation. E168 and E182 were predicted consensus methylation sites, but E175 is methylated on an HE pair. To determine the roles of these sites *in vivo*, we substituted each methylatable glutamate with either an aspartate or an alanine residue and determined the impact of the point mutants on single cell reversals, swarming and fruiting body formation. Single, double and triple methylation site mutants revealed that each site played a unique role in *M. xanthus* behaviour and that the pattern of receptor methylation determined receptor activity. This work also shows that methylation can both activate and inactivate the receptor.

## Introduction

*Myxococcus xanthus* is a Gram-negative bacterium that has a complex life cycle that involves vegetative swarming, predation and fruiting body formation (Reichenbach, 1999; Shimkets, 1999; Kaiser, 2003; 2004; Berleman *et al*., 2006). These behaviours require motility on solid surfaces: S-motility powered by Type IV pili (Wu and Kaiser, 1995) moves cells in groups and A-motility powered by unidentified motor proteins and putative adhesion complexes moves single cells (Mignot, 2007). To achieve directed movements, *M. xanthus* cells periodically reverse so that the leading pole becomes the lagging pole. The frequency of cell reversals is controlled by the *frz* chemosensory pathway and is important for directed cell movements (Blackhart and Zusman, 1985). *frz* mutants (Δ*frzA*, Δ*frzB,* Δ*frzCD,* Δ*frzE,* Δ*frzF*) rarely reverse and are therefore defective in swarming and fruiting body formation, forming frizzy aggregates instead of fruiting bodies on starvation media (Zusman, 1982). In contrast, some constitutively signalling *frz* mutants hyper-reverse, forming very compact colonies with little cell spreading (Blackhart and Zusman, 1985; Bustamante *et al*., 2004).

Frz proteins are homologous to bacterial chemotaxis proteins (McBride *et al*., 1989; Zusman *et al*., 2007). FrzCD, a cytoplasmic methyl-accepting chemotaxis protein (MCP) homologue, lacks the transmembrane and periplasmic domains common to most MCPs and has in its place a unique N-terminal domain. In contrast, the C-terminal domain of FrzCD is similar to other MCPs and contains potential methylation sites (Astling *et al*., 2006). *In vitro* analysis has shown that FrzCD interacts with FrzE, a histidine kinase (CheA)-response regulator fusion protein by means of two CheW-like proteins, FrzA and FrzB (Astling, 2003). When stimulated, FrzE autophosphorylates and transfers a phosphoryl group to the dual response regulator FrzZ, triggering cell reversals for both the A- and S-motility systems (Inclán *et al*., 2007).

It is not known how FrzCD receives signals as it lacks the usual signal-binding domain common to most MCPs. Furthermore, *frzCD* N-terminal domain deletion mutants show only minor defects in behaviour (Bustamante *et al*., 2004). Previously, it was hypothesized that a signal input to the Frz pathway may involve differential methylation of particular sites on the receptor (Igoshin *et al*., 2004; Astling *et al*., 2006). Astling *et al*. (2006) identified several putative methylation sites based on sequence in comparison with known *Escherichia coli* methylation sites. They systematically mutated these sites, substituting each EE, QQ, QE and EQ pair with an alanine pair (AA). This work suggested that FrzCD receptor activity could be turned on or off depending on the site that was methylated.

Based on this work, we hypothesized that differential methylation of FrzCD may be mediated by FrzF, a methyltransferase (CheR) homologue (McCleary *et al*., 1990) that contains an additional domain with three tetra trico-peptide repeats (TPRs) (Shiomi *et al*., 2002). In other organisms, TPRs have been shown to mediate protein–protein interactions (Blatch and Lassle, 1999). A Basic Local Alignment Search Tool (blast) (Altschul *et al*., 1990) analysis of all sequenced bacterial genomes revealed that dozens of bacterial species possess putative methyltransferases with one or more TPRs, including several within the α-proteobacteria, β-proteobacteria, δ-proteobacteria and high GC-rich Gram-positive bacteria. In *M. xanthus*, CheR4 and CheR6 are each predicted to contain one TPR (Scott, 2008). However, to our knowledge, no function has yet been attributed to TPRs in methyltransferases.

To investigate the role of the TPR containing domain in FrzCD methylation, we used full-length FrzF and FrzF lacking the TPRs (FrzF^CheR^) to methylate FrzCD *in vitro*. We found that indeed the TPRs of FrzF negatively regulate FrzCD methylation. We used mass spectrometry to identify the methylated sites and site-directed mutagenesis to determine the function of these sites *in vivo*. This work showed that each FrzCD methylation site played specific roles in cell motility and behaviour.

## Results

### Methylation of FrzCD *in vitro* using purified FrzF and FrzF^CheR^

**Fig. 1:**
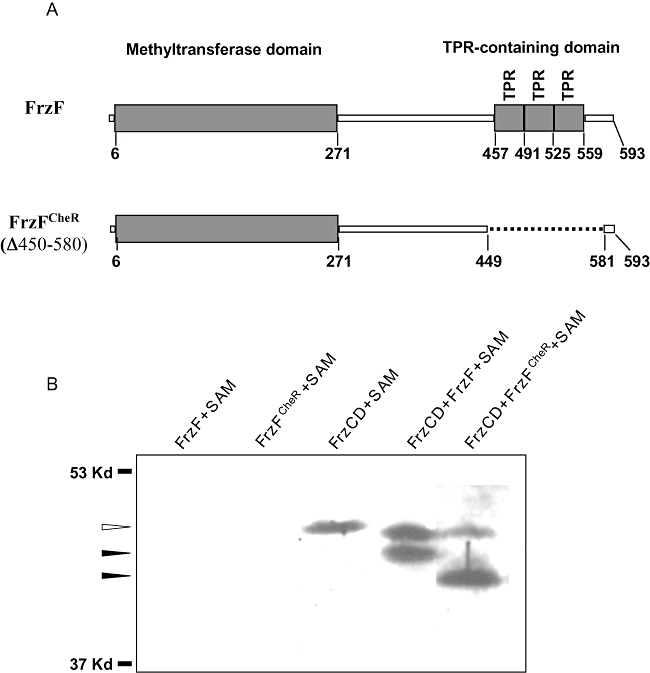
Methylation of FrzCD by FrzF and FrzF^CheR^*in vitro*. A. Cartoons show the domain organization of the wild-type FrzF protein and the FrzF^CheR^ protein, which lacks FrzF amino acids 450–580. TPR indicates a tetra-trico peptide repeat. Numbers refer to the amino acid position in full-length FrzF. B. His-tagged FrzCD was expressed and purified from *E. coli* and incubated *in vitro* with purified FrzF or FrzF^CheR^ and S-adenosyl methionine (SAM) for 4 h at 32°C. Following the reaction, FrzCD was analysed by SDS-PAGE and Western immunoblotting using purified α-FrzCD antibodies. The white arrowhead shows the mobility of unmethylated FrzCD; the black arrowheads indicate methylated FrzCD. The position of the molecular weight markers is indicated on the left.

Previous work showed that methylation of FrzCD is required for swarming and fruiting body formation in *M. xanthus* and that methylation is mediated by FrzF (McBride *et al*., 1989; McCleary *et al*., 1990). FrzF is a complex methyltransferase that contains an N-terminal domain with 31% sequence identity (83 of 271 amino acids) to the methyltransferase (CheR) of *E. coli* and a C-terminal domain with three TPRs (Fig. 1A). Because of its high homology to CheR, the N-terminal domain of FrzF was assumed to have methyltransferase activity, but the function of the C-terminal domain was unknown. To determine the activities of full-length FrzF and FrzF^CheR^ (FrzF lacking its TPR domains), we cloned His-tagged *frzCD, frzF* and *frzF*^*CheR*^ in expression vectors and purified the respective proteins from *E. coli*. FrzCD purified from *E. coli* was unmethylated (Fig. 1B, lane 3), indicating that the *E. coli* methyltransferase does not methylate FrzCD. To methylate FrzCD *in vitro*, we incubated FrzCD and the methyl donor S-adenosyl methionine (SAM) with either FrzF or FrzF^CheR^. The reactions were monitored by Western immunoblot analysis using purified anti-FrzCD antibodies as methylated FrzCD migrates faster than unmethylated FrzCD in this SDS-PAGE system (McCleary *et al*., 1990). We found that that both FrzF and FrzF^CheR^ were able to methylate FrzCD in the presence of SAM (Fig. 1B), but that they produced different FrzCD methylation patterns. FrzF^CheR^ produced a faster migrating band of FrzCD (Fig. 1 lane 5) than FrzF (Fig. 1 lane 4). Neither FrzF nor FrzF^CheR^ were able to methylate FrzCD without SAM (data not shown). As methylated receptors migrate faster than unmethylated receptors in this gel system, we hypothesized that the faster migrating band observed with the FrzF^CheR^ sample represented a more methylated species of FrzCD.

### Identifying the methylated residues of FrzCD

As the mobility of FrzCD on polyacrylamide gels does not give us quantitative data on methylation patterns, we analysed the *in vitro* methylated FrzCD by mass spectrometry. We prepared methylated FrzCD samples as described above by incubating purified FrzCD, SAM and either FrzF or FrzF^CheR^; as a control, we also prepared non-methylated FrzCD in the same way except that we omitted FrzF. To generate peptides of the optimal size for mass spectrometry, we digested the methylated and unmethylated FrzCD samples with trypsin, chymotrypsin or GluC proteases. The combined tandem mass spectrometry (MS/MS) results accounted for 92.3% total sequence coverage including 100% sequence coverage of the C-terminus of FrzCD (amino acids 136–437), predicted to contain all putative methylation sites (Fig. S1).

A comparison between spectra of tryptic FrzCD peptides methylated by FrzF and by FrzF^CheR^ revealed that FrzF methylated FrzCD on a single glutamate residue and that FrzF^CheR^ methylated FrzCD on three residues (based on accurate precursor mass measurement and MS/MS data) within the peptide L148-K184. This peptide sequence includes five glutamate residues, all representing possible sites of methylation. However, while fragmentation was sufficient for the identification of the peptide, as well as the presence of methylation, it was not adequate to obtain the sequence coverage necessary to ascertain the specific sites of methylation (data not shown). To address this, FrzCD was digested with the chymotrypsin and GluC proteases. Chymotrypsin allowed us to determine that FrzCD was methylated by FrzF on E182 (Table 1, Fig. S2). Specifically, the b-ion with the value of 1372.3 indicates that there is a methylated glutamate on the N-terminal side of residue L183 and the b-ion with the value of 1301.1 indicates that no residue is methylated to the N-terminal side of E182. Thus, the only residue that can be methylated on this peptide is E182. However, we found that the peptides were not always methylated. For instance, two of the nine _157_AASTQHETSSTEQAAA IHETTATMEEL_183_ peptides analysed showed methylation on site E182 (Table 1, Fig. S2, and data not shown). No methylation was seen on sites E168 or E175 on any of the nine peptides. MASCOT scores for these nine peptides were all greater than 24 indicating that these results are highly significant.

**Table 1:** Ions from MS/MS spectrum of a chymotryptic fragment^a^ of FrzCD show FrzF methylates site E182.

AA^b^	Theoretical^c^ b-ions^d^	Observed^e^ b-ions	Theoretical y-ions^f^	Observed y-ions
I173	842.9 (2+)	843.0 (2+)		
H174				
E175				
T176	1026.5 (2+)	1026.3 (2+)		
T177			824.4	824.0
A178	1112.5 (2+)	1112.5 (2+)	723.3	723.1
T179	1163.0 (2+)	1163.0 (2+)	652.3	652.1
M180	1236.6 (2+)	1236.9 (2+)	551.2	551.1
E181	1301.1 (2+)	1301.0 (2+)		
E182#	1372.6 (2+)	1372.3 (2+)		
L183				

a No ions were observed for residues _157_AASTQHETSSTEQAAA172 of the chymotryptic peptide _157_AASTQHETSSTEQAAAIHETTATMEEL_183_ (Fig. S2), so these residues were removed from the left column for simplicity.

b Amino acids are indicated by their one letter code; numbers represent their position in FrzCD. The # symbol represents an amino acid that is methylated.

c Theoretical ions are calculated by dividing the predicted mass of an ion by the ion's charge, m/z = (M + nH^+^)/n.

d A b-ion is an N-terminal charged fragment generated after ion activation causes a peptide bond to break.

e Observed ions were found by digesting FrzCD with chymotrypsin and using tandem MS/MS.

f A y-ion is a C-terminal charged fragment generated after ion activation causes a peptide bond to break.

All ions are 1+ charged unless otherwise indicated in parentheses.

We found that FrzF^CheR^ methylated FrzCD on residues E175 and E182 by using a chymotrypsin digest (Table 2, Fig. S3A). Specifically, b-ion 849.7 showed that the methylated glutamate was on the C-terminal side of residue I173 and b-ion 1040.6 showed that the methylated glutamate was on the N-terminal side of T176. Thus, E175 must be methylated as it is the only glutamate between residues I173 and T176. b-ion 1315.2 showed that another methylated glutamate was located on the C-terminal side of residue E181. Therefore, E182 was a site of methylation (Table 2, Fig. S3A). Similar to our FrzF data, we found that the FrzF^CheR^ methylated FrzCD peptides were not always fully methylated. For instance, one of the three _157_AASTQHETSSTEQA AAIHETTATMEEL_183_ peptides analysed showed methylation on sites E175 and E182 (Table 2, Fig. S3A). The remaining two peptides showed methylation only on site E182 (Data not shown). MASCOT scores for these three peptides were all greater than 24 indicating that these results are highly significant.

**Table 2:** Ions from a MS/MS spectrum of a chymotryptic fragment^a^ of FrzCD show FrzF^CheR^ methylates sites E175 and E182.

AA^b^	Theoretical^c^ b-ions^d^	Observed^e^ b-ions	Theoretical y-ions^f^	Observed y-ions
I173	849.9 (2+)	849.7 (2+)		
H174				
E175#				
T176	1040.5 (2+)	1040.6 (2+)		
T177	1091.0	1091.4	824.4	824.2
A178	1126.5 (2+)	1126.6 (2+)	723.3	722.9
T179	1177.0 (2+)	1177.1 (2+)	652.3	652.0
M180	1250.6 (2+)	1251.0 (2+)	551.2	551.0
E181	1315.1 (2+)	1315.2 (2+)		
E182#	1372.6 (2+)	1372.3 (2+)		

a No ions were observed for residues _157_AASTQHETSSTEQAAA172 of the chymotryptic peptide _157_AASTQHETSSTEQAAAIHETTATMEEL_183_ (Fig. S3A), so these residues were removed from the left column for simplicity.

b Amino acids are indicated by their one letter code; numbers represent their position in FrzCD. The # symbol represents an amino acid that is methylated.

c Theoretical ions are calculated by dividing the predicted mass of an ion by the ion's charge, m/z = (M + nH^+^)/n.

d A b-ion is an N-terminal charged fragment generated after ion activation causes a peptide bond to break.

e Observed ions were found by digesting FrzCD with chymotrypsin and using tandem MS/MS.

f A y-ion is a C-terminal charged fragment generated after ion activation causes a peptide bond to break.

All ions are 1+ charged unless otherwise indicated in parentheses.

To find the third site methylated by FrzF^CheR^, we used a GluC digest (Table 3, Fig. S3B). The b-ion 648.2 showed that a glutamate was methylated on the N-terminal site of Q169 and the y-ion 1546.4 showed that a glutamate was methylated on the C-terminal side of T167. In all seven _164_TSSTEQAAAIHE175 peptides analysed, site E168 was shown to be methylated (data not shown). In one out of four the _164_TSSTEQAAAIHETTATME181 peptides analysed, both E168 and E175 were found to be methylated (Fig. S3B). In all eight of the _169_QAAAIHETTATME181 peptides site E175 was methylated (data not shown). MASCOT scores for these peptides were all greater than 24 indicating that these results are highly significant.

**Table 3:** Ions from an MS/MS spectrum of a GluC digested fragment^a^ show FrzF^CheR^ methylates FrzCD on sites E168 and E175.

AA^b^	Theoretical^c^ b-ions^d^	Observed^e^ b-ions	Theoretical y-ions^f^	Observed y-ions
E168			1546.7	1546.4
Q169	648.3	648.2	1403.6	1403.4
A170	719.3	719.2	1275.6	1275.3
A171			1204.6	1204.4
A172	861.4	861.2	1133.5	1133.3
I173	974.5	974.3	1062.5	1062.3
H174	1111.5	1111.3	949.4	949.3
E175#	1254.6	1255.3		
T176	1355.6	1355.4	669.3	669.1
T177	1456.7	1456.4	568.2	568.1
A178	1527.7	1527.5	467.2	467.1
T179	1628.8	1628.5	396.1	396.1
M180			295.1	295.1

a No ions were observed for residues _164_TSST167 of the GluC-generated fragment _164_TSSTEQAAAIHETTATME181 (Fig. S3B), so these residues were removed from the left column for simplicity.

b Amino acids are indicated by their one-letter code; numbers represent their position in FrzCD. The # symbol represents an amino acid that is methylated.

c Theoretical ions are calculated by dividing the predicted mass of an ion by the ion's charge, m/z = (M + nH^+^)/n.

d A b-ion is an N-terminal charged fragment generated after ion activation causes a peptide bond to break.

e Observed ions were found by digesting FrzCD with chymotrypsin and using tandem MS/MS.

f A y-ion is a C-terminal charged fragment generated after ion activation causes a peptide bond to break.

All ions are 1+ charged.

The sample without FrzF was not methylated at any site (Table S1, Fig. S4, and data not shown). None of the 14 copies of the _157_AASTQHETSSTEQA AAIHETTATMEEL_183_ peptide showed methylation (Table S1 and Fig. S2). MASCOT scores for these 14 peptides were all greater than 24 indicating that these results are highly significant.

In summary, mass spectrometry showed that *in vitro* FrzF methylates FrzCD on one site (E182) and that FrzF^CheR^ methylates FrzCD on three sites (E168, E175 and E182). This would suggest that the TPR domain of FrzCD is a regulatory domain that inhibits the methyltransferase activity of FrzF at two specific sites, E168 and E175.

### Isolation and characterization of methylation site point mutants

To learn the function of these three FrzCD methylation sites, we constructed FrzCD methylation point mutants where we replaced a methylatable glutamate residue with either an aspartate or an alanine residue. These substitutions have been used in other bacteria to mimic unmethylated and methylated glutamates, respectively, as aspartate residues, which cannot be methylated, maintain the negative charge of a glutamate residue and alanine residues are neutrally charged, similar to methylated glutamates (Nowlin *et al*., 1988; Park *et al*., 1990; Shapiro and Koshland, 1994).

**Fig. 2:**
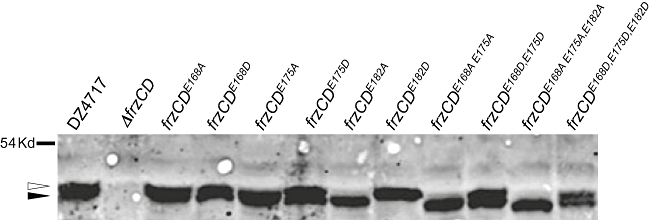
FrzCD methylation point mutants are stably expressed. An immunuoblot of vegetative cell extracts from the methylation point mutants probed with the α-FrzCD antibody. Thirty micrograms of total protein from whole cell extracts were loaded per lane. The white arrowhead shows the mobility of unmethylated FrzCD; the black arrowhead indicates methylated FrzCD. DZ4717 (*6His::frzCD*) is the positive control strain used in this study. All point mutations were made in the DZ4717 background.

FrzCD methylation site point mutants were generated by PCR, cloned into the plasmid pCT2, and integrated into the non-essential *crtB* locus of the *M. xanthus* Δ*frzCD* strain. We expressed *frzCD* under control of the *tet* promoter by growing cells in the presence of anhydrotetracycline (Mignot *et al*., 2007). As a positive control we made strain DZ4717, which contains the non-mutated *frzCD* gene in the *crtB* locus under the control of the *tet* promoter. We found that DZ4717 displayed phenotypes that were comparable to that of the wild-type strain, DZ2 (Fig. S5). To ensure that all strains expressed the FrzCD protein, we examined each strain by immunoblot analysis using the anti-FrzCD antibody. Figure 2 shows that FrzCD was expressed at the same level in all strains and that any changes in phenotype were not due to altered expression.

**Fig. 3:**
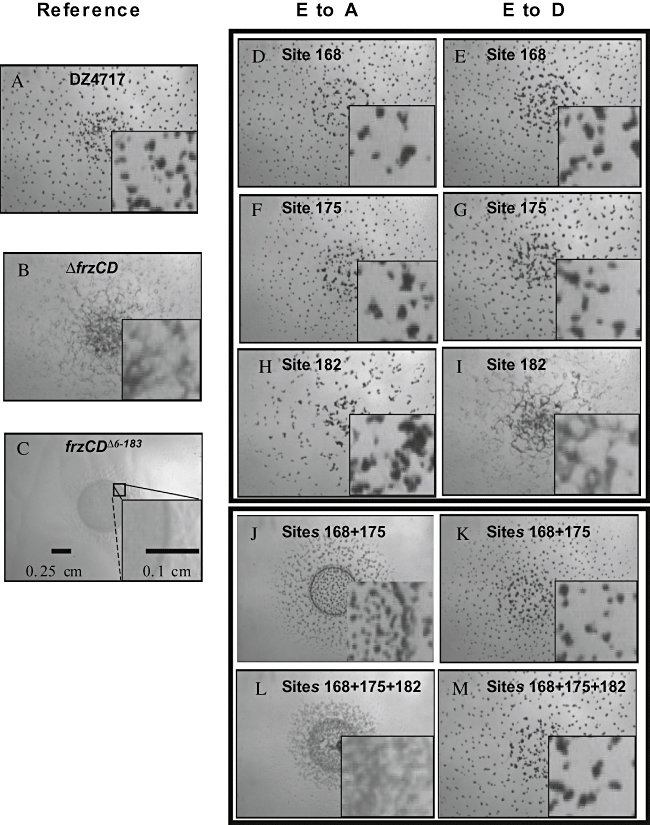
Effect of FrzCD E to A and E to D methylation site mutations on aggregation and fruiting body formation. FrzCD methylation site glutamates (E) 168, 175 and 182 were changed to alanine (A) or aspartate (D) residues by site directed mutagenesis as described in *Experimental procedures*. Cells were spotted at 4 × 10^9^ cells ml^−1^ on CF fruiting agar and incubated for 4 days at 32°C. Reference strains (DZ4717 (*6His::frzCD*), Δ*frzCD*, and *frzCD*Δ*6*^−*183*^) are shown in A–C. FrzCD E to A mutants are shown in the middle column (D, F, H, J and L) and FrzCD E to D mutants are shown in the right column (E, G, I, K and M). The small corner inset in each picture is a 7 × magnification of a portion of the original.

**Fig. 4:**
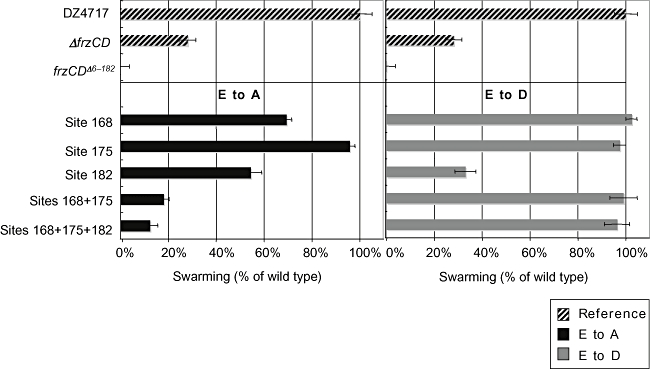
Effect of FrzCD E to A and E to D methylation site mutations on vegetative swarming. Reference strains [DZ4717 (*6His::frzCD*), Δ*frzCD* and *frzCD*^Δ*6*–*183*^] are shown at the top (striped bars), FrzCD E to A mutants are shown in the left panel (dark grey), and FrzCD E to D mutants are shown in the right panel (light grey). The horizontal axis represents the relative distance swarmed by each strain compared with wild type. Error bars represent the standard deviation of the mean. Five microlitres of 4 × 10^9^ cells ml^−1^ were spotted on CYE nutrient plates containing 0.4% agar and swarm expansion was measured after 3 days incubation at 32°C. Data shown are from two independent experiments with a total of seven measurements per strain.

We then determined the effects of mutating the methylation sites on *M. xanthus* fruiting body formation (Fig. 3) and swarming (Fig. 4), phenotypes that require a functioning Frz pathway. For reference, we also examined the phenotypes of the hypo-reversing Δ*frzCD* mutant and the hyper-reversing *frzCD*^Δ*6*–*183*^ mutant. As shown previously (Bustamante *et al*., 2004), the Δ*frzCD* control strain exhibited reduced swarming on rich media, formed tangled aggregates instead of fruiting bodies on starvation media, and reversed infrequently. In contrast, the *frzCD*^Δ*6*–*183*^ control strain did not swarm on rich media, was unable to form aggregates on starvation media, and reversed much more frequently as single cells than wild type (Figs 3 and 4; Table 4). The methylation point mutants displayed varied phenotypes: some behaved like wild type, whereas others displayed Δ*frzCD*, *frzCD*^Δ*6*–*183*^ or intermediate phenotypes.

**Table 4:** Effect of methylation site mutations on single cell reversals.

Strain	Average reversals in 30 min (# cells)	Average reversals in 30 min E to D (# cells)
DZ4717^a^	1.58 (84)	
Δ*frzCD*	0.20 (59)	
*frzCD*Δ^*6*−*183*^	12.14 *(*24)	
Site 168	1.67* (49)	1.57* (74)
Site 175	0.82 (44)	0.25 (58)
Site 182	0.58 (29)	0.22 (34)
Sites 168 + 175	6.25 (70)	1.34* (59)
Sites 168 + 175 + 182	4.63 (56)	1.18* (42)

a DZ4717 has the same reversal frequency as wild type (see Fig. S5).

Asterisks indicate that reversals are statistically the same as DZ4717 (Student's *t*-test). Values that were statistically different from DZ4717 had *P*-values less than 0.005 (Student's *t*-test).

- The most severe single mutant phenotypes were observed when we eliminated methylation at E182. The *frzCD*^*E182D*^ mutant, similar to Δ*frzCD*, showed reduced swarming (Fig. 4), formed tangled aggregates instead of fruiting bodies (Fig. 3I), and had a severely reduced single cell reversal frequency when compared with DZ4717 (Table 4). The *frzCD*^*E182A*^ mutant displayed an intermediate swarming phenotype between DZ4717 and the *frzCD*^*E182D*^ mutant (Fig. 4), was able to form fruiting bodies (Fig. 3H), but still showed a reduced single cell reversal frequency (Table 4). Thus, FrzCD methylation site 182 is important for both social behaviours and single cell reversals.
- Single mutations in E175 or E168 resulted in less severe defects than single mutations in site E182. E to A or E to D mutations at site 175 allowed cells to swarm like DZ4717 (Fig. 4) and to form fruiting bodies (Figs 3F,G). Yet both of these mutants showed a decrease in reversal frequency when compared with DZ4717 (Table 4). The *frzCD*^*E168D*^ mutant displayed no obvious defects. While the *frzCD*^*E168A*^ mutant displayed a 30% decrease in swarming (Fig. 4), it showed normal single cell and fruiting body behaviour. Thus, site E175 is required for single cell reversal frequency, but neither site E168 nor E175 must be methylated for proper fruiting body formation.

In *E. coli,* methylation site mutants display additive phenotypes; defects increase in severity as additional methylation sites are mutated (Shapiro *et al*., 1995). To test if methylation site mutations resulted in additive defects in *M. xanthus*, we constructed double and triple methylation site point mutants. We expected the double and triple mutants to display more severe defects than the single mutants. However, we found that the *frzCD*^*E168D,E175D*^ and *frzCD*^*E168D,E175D,E182D*^ mutants displayed DZ4717 phenotypes under all conditions tested (Fig. 3K and M, Fig. 4, Table 4). This was surprising as the *frzCD*^*E175D*^ and the *frzCD*^*E182D*^ mutants displayed reduced single cell reversal frequencies compared with DZ4717. These results suggest that the overall number of methylatable FrzCD residues is not crucial, but rather the pattern of FrzCD methylation is the determining factor for receptor function.

We were also surprised to find that the *frzCD*^*E168A,E175A*^ and *frzCD*^*E168A,E175A,E182A*^ mutants displayed severe defects under all conditions tested, as the single site mutants, E168A and E175A displayed no or subtle defects. These mutants swarmed less than Δ*frzCD* (Fig. 4), hyper-reversed compared with DZ4717 (Table 4), and formed smaller and less dispersed fruiting bodies than DZ4717 during development (Fig. 3J and L). The *frzCD*^*E168A,E175A*^ and *frzCD*^*E168A,E175A,E182A*^ mutants displayed phenotypes that were intermediate between DZ4717 and the hyper-reversing *frzCD*^Δ*6*–*183*^ mutant.

## Discussion

FrzCD, the principal MCP for the Frz chemosensory pathway, plays a central role in regulating cellular motility and behaviour in *M. xanthus*. Previous studies showed that methylation of FrzCD by FrzF is critical to function of FrzCD, as cells lacking FrzF, the unusual TPR domain containing methyltransferase, rarely reverse and are unable to swarm, form fruiting bodies, or move towards nutrients (Blackhart and Zusman, 1985; Bustamante *et al*., 2004). We were curious to learn the role of the TPRs in FrzF and how methylation regulates FrzCD receptor activity.

**Fig. 5:**
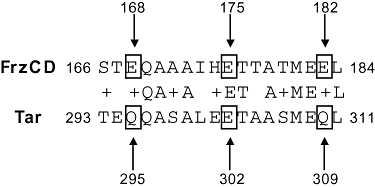
Similarities between the methylation site sequences of *M. xanthus* FrzCD and *E. coli* Tar. The FrzCD peptide containing identified methylated residues is shown aligned with a methylated peptide from the *E. coli* receptor, Tar. Residues that are identical or that share similar charges are indicated on the second row by a letter (amino acid) or ‘+’ respectively. Sites of methylation are indicated by a box and an arrow. Numbers indicate amno acid position in FrzCD.

By analysing *in vitro* methylated FrzCD by mass spectrometry, we found that FrzF methylates FrzCD on one residue, E182, and that FrzF^CheR^ methylates FrzCD on three residues, E168, E175 and E182. When we compared the methylated residues of FrzCD with methylated residues of the *E. coli* MCP, Tar, we found both similarities and differences (Fig. 5). Both FrzCD and Tar have methylation sites spaced seven residues apart. It has been shown that methylation sites spaced at seven residue intervals are located on the same face of the α-helix and are accessible to the methyltransferase (Terwilliger *et al*., 1986).

Several differences exist between the methylation sequences of Tar and FrzCD. Tar is methylated on glutamine (Q) or glutamate (E) residues that are located within QQ, EE, QE or EQ methylation pairs and methylation occurs on the second residue of the methylation pair (Kehry and Dahlquist, 1982; Terwilliger *et al*., 1983; Terwilliger and Koshland, 1984). In FrzCD, only site E182 conforms precisely to the *E. coli* paradigm; it is methylated on the second residue of an EE methylation pair. Site E175 is the most unusual of the FrzCD methylation sites; it is not located in a typical methylation pair. Site E175 is flanked instead by histidine (H) and threonine (T) residues (Fig. 5). This is only the second instance of a receptor being methylated outside of a typical methylation pair. In *Thermotoga maritima*, the receptor TM0429c is methylated on the glutamate in a TE pair (Perez *et al*., 2006). FrzCD site E168 is also somewhat atypical because methylation is occurring on the first residue of a methylation pair (Fig. 5). Although unusual, other receptors have been shown to be methylated on the first residue of a methylation pair (Perez *et al*., 2006). We found it interesting that FrzCD site E182, which conforms precisely to the *E. coli* consensus sequence, was methylated by both FrzF and FrzF^CheR^*in vitro*, but that sites E168 and E175, which differ from the *E. coli* consensus sequence, were only methylated by FrzF^CheR^. It is possible that methylation preferentially occurs on sites that adhere more closely to the methylation consensus sequence found in *E. coli*.

To learn how methylation affects the activity of FrzCD we constructed mutations in each of the identified methylation sites. A previous study had mutated each QQ, EE, EQ and QE pair in FrzCD to a double AA (Astling *et al*., 2006). Because FrzCD sites E168 and E182 were located within EQ and EE pairs, respectively, the FrzCD^E168AQ169A^ and FrzCD^E181AE182A^ mutants were constructed, previously. Astling *et al*. (2006) found by analysing single cell reversals that the FrzCD^E168AQ169A^ mutant appeared to hyperactivate FrzCD, whereas the FrzCD^E181AE182A^ mutant resulted in a hypoactive FrzCD signally state. Although these results were promising, we were concerned that the double AA mutation may result in a more severe phenotype than a single mutant. Additionally, we identified a FrzCD methylation site that was located outside of a QQ, EE, QE, EQ methylation pair and were interested to learn its function. We were also interested in learning the phenotype of both the gain and loss of methylation at each site; therefore, we constructed mutants where we replaced each methylatable glutamate with an alanine to mimic methylation or an aspartate to mimic demethylation (Nowlin *et al*., 1987; Shapiro and Koshland, 1994).

We found that site 182 (the site methylated by both FrzF and FrzF^CheR^) displayed the most profound phenotypes of the single mutants. In fact, the *frzCD*^*E182D*^ mutant behaved similarly to the Δ*frzCD* mutant suggesting that methylation at this residue is critical for activating the Frz pathway. Furthermore, the *frzCD*^*E182D*^ mutant phenotype also resembled the Δ*frzE* and the *frzE*^*H49A*^ (kinase dead) mutant phenotypes suggesting that FrzCD^*E182D*^ may inhibit the histidine kinase activity of FrzE. The severe mutant phenotype seen in the *frzCD*^*E182D*^ strain may be due to loss of methylation at other nearby methylation sites. In *E. coli*, replacing a methylatable E or Q residue with a D residue has been shown to prevent methylation at nearby sites (Shapiro *et al*., 1995). This appears to hold true for the *frzCD*^*E182D*^ mutant. We do not see any FrzCD methylation in *M. xanthus* in the *frzCD*^*E182D*^ mutant (Fig. 2). As the *frzCD*^*E182D*^ mutant displayed a more severe phenotype than either the *frzCD*^*E168D,E175D,E182D*^ or *frzCD*^*E168D,E175D*^ mutants, we concluded that the *frzCD*^*E182D*^ mutant phenotype was not due to loss of methylation at sites E168 or E175. It is likely that the *frzCD*^*E182D*^ mutant lacks methylation at unidentified sites. We believe that additional FrzCD methylation sites exist because the *frzCD*^*E168D,E175D,E182D*^ mutant still displays a methylated band of FrzCD (Fig. 2). We anticipate that several additional methylation sites may be uncovered when glutamine residues are converted to glutamates by a methylesterase *in vivo* (Stock and Koshland, 1978). Additionally, some methylation sites may only be modified *in vivo* under certain physiological conditions.

Mutations in FrzCD site E175 resulted in less severe mutant phenotypes compared with mutations in FrzCD site E182. Strains containing an aspartate or an alanine at site 175 displayed a decrease in single cell reversal frequency, but showed no obvious defect in social swarming or fruiting body development. These results suggest that site E175 is critical for coordinating single cell behaviour, but is dispensable for groups of cells. This is interesting because it suggests that FrzCD is able to regulate single cell behaviour and social behaviour independently.

FrzCD site 168 appears to play a role in regulating social swarming. The *frzCD*^*E168A*^ mutant displayed a reduction of social swarming, but did not have an obvious defect in fruiting body formation and single cells reversed normally. The *frzCD*^*E168D*^ mutant did not display any mutant phenotypes. Thus, site E168 appears to be a minor regulator of FrzCD when methylated alone. However, when both FrzCD sites E168 and E175 were replaced with alanines, *M. xanthus* cells displayed profound affects; cells were unable to swarm, they were defective in fruiting body formation, and single cells hyper-reversed. Thus, simultaneous methylation at these two sites seems to activate FrzCD. It was interesting that the *frzCD*^*E168D,E175D*^ mutant did not display any mutant phenotypes. Thus, the mutation at site 168 can rescue the hypo-reversing phenotype of the *frzCD*^*E175D*^ mutant.

In sum, we found that the TPRs of FrzF inhibit its ability to methylate FrzCD *in vitro*. Additionally, each site of FrzCD methylation plays a unique role in regulating FrzCD activity. Third, FrzCD appears to be able to control single cell and social behaviours independently. Last, our results confirm previous work by Astling *et al*. (2006), which suggested that FrzCD methylation could both turn on and off receptor activity and that the pattern of methylation determines the activity of FrzCD, not the quantity of methylation.

Based on our current knowledge of the Frz pathway, we have proposed a model that addresses a putative input into FrzCD, the regulation of FrzCD activity, and how FrzCD activity regulates downstream signalling and cell behaviour. We believe that one input into FrzCD can be the methylation of FrzCD by FrzF. FrzF may be controlled by its TPRs. As FrzF^CheR^ methylates FrzCD more than FrzF *in vitro*, it is possible that the TPRs prevent the methyltransferase domain from methylating specific residues of FrzCD (such as E168 and E175) by forming a physical barrier between the methyltransferase domain and certain binding sites on FrzCD. To relieve this inhibition, the TPRs may bind to another protein. This binding may place the TPRs in a conformation in which they no longer impede the methyltransferase domain, thus allowing FrzF to methylate additional FrzCD residues. We hypothesize that a FrzF TPR-binding partner is up-regulated or activated as cells proceed through development because FrzCD methylation has been shown to increase as *M. xanthus* cells develop (McBride and Zusman, 1993). Additionally, we propose that the TPR-binding partner is upregulated by AsgA, CsgA, FruA and DevT and downregulated by RodK because FrzCD methylation is reduced in *asgA*, *csgA*, *fruA* and *devT* mutants, and increased in *rodK* mutants (Sogaard-Andersen and Kaiser, 1996; Geng *et al*., 1998; Boysen *et al*., 2002; Rasmussen *et al*., 2005).

The second part of our model explains how the activity of FrzCD can be controlled through methylation. We propose that methylation can both turn on and turn off FrzCD activity. For instance, if FrzCD is methylated on one site (E182) its activity is inhibited, whereas if it is methylated on additional residues (E168 and E175) its activity is activated. These hypotheses are based on our FrzCD methylation point mutant data where mutations in site E182 were similar to a deletion of FrzCD and mutations that mimicked methylation at both 168 and 175 resembled a ‘hyperactive’ *frzCD* mutant (*frzCD*^Δ*6−183*^).

The final part of our model involves how FrzCD activity regulates downstream proteins. It has been proposed that the CheA homologue, FrzE, stimulates cellular reversals by autophosphorylating and transferring a phosphoryl group to the dual receiver domain containing protein, FrzZ (McCleary and Zusman, 1990; Inclán *et al*., 2007). We propose that certain methylation sites lead to an active form of FrzCD and this stimulates FrzE kinase activity, which leads to an increase in cellular reversals. Conversely, methylation states of FrzCD that inhibit activity result in a reduction of FrzE kinase activity and a reduction in cellular reversals.

## Experimental procedures

### Bacterial strains and culture conditions

The strains used in this study are listed in Table 5. *M. xanthus* was grown in CYE medium, which contains 10 mM morpholinepropanesulphonic acid (MOPs, pH 7.6), 1% (w/v) Bacto Casitone (BD Biosciences), 0.5% Bacto yeast extract and 4 mM MgSO_4_ (Campos *et al*., 1978). For *M. xanthus* swarming phenotypes 5 μl of cells at 4 × 10^9^ cells ml^−1^ were spotted on CYE plates containing 0.5% Bacto agar (BD Biosciences). For developmental phenotypes 5 μl of cells at 2 × 10^9^ cells ml^−1^ were spotted on CF agar which contains 1.5% Bacto agar, 10 mM MOPs (pH 7.6), 0.015% Bacto Casitone, 8 mM MgSO_4_, 1 mM KH_2_PO_4_, 0.02% NH_4_SO_4_, 0.2% sodium citrate and 0.1% pyruvate (Hagen *et al*., 1978).

**Table 5:** Strains and plasmids used in this study.

Strain or plasmid	Relevant feature	Source
*M. xanthus*		
DZ2	Wild type	Laboratory collection
VB197b	*frzCD*^Δ*6−183*^	Bustamante *et al*. (2004)
DZ4480	Δ*frzCD*	Bustamante *et al*. (2004)
DZ4717	Δ*frzCD*, *6His::frzCD* integrated in *crtB*	This study
DZ4707	Δ*frzCD 6His::frzCD*^*E168A*^ integrated in *crtB*	This study
DZ4708	Δ*frzCD 6His::frzCD*^*E175A*^ integrated in *crtB*	This study
DZ4709	Δ*frzCD 6His::frzCD*^*E182A*^ integrated in *crtB*	This study
DZ4710	Δ*frzCD 6His::frzCD*^*E168D*^ integrated in *crtB*	This study
DZ4711	Δ*frzCD 6His::frzCD*^*E175D*^ integrated in *crtB*	This study
DZ4712	Δ*frzCD 6His::frzCD*^*E182D*^ integrated in *crtB*	This study
DZ4713	Δ*frzCD 6His::frzCD*^*E168A E175A*^ integrated in *crtB*	This study
DZ4714	Δ*frzCD 6His::frzCD*^*E168D E175D*^ integrated in *crtB*	This study
DZ4718	Δ*frzCD 6His::frzCD*^*E168A E175A E182A*^ integrated in *crtB*	This study
DZ4719	Δ*frzCD 6His::frzCD*^*E168D E175D 182D*^ integrated in *crtB*	This study
*E. coli*		
Top10	General cloning strain	Invitrogen
Tuner	Protein expression strain	Novagen
Plasmids		
pET28a	Expression plasmid	Novagen
pAS201	pET28a with *6His::frzF*^*cheR*^	This study
pCT2	*M. xanthus* genomic integration plasmid	Mignot *et al*. (2007b)
pAS210	pCT2 with *6His::FrzCD*	This study
pAS211	pCT2 with *6His::frzCD*^*E168A*^	This study
pAS212	pCT2 with *6His::frzCD*^*E175A*^	This study
pAS213	pCT2 with *6His::frzCD*^*E182A*^	This study
pAS214	pCT2 with *6His::frzCD*^*E168D*^	This study
pAS215	pCT2 with *6His::frzCD*^*E175D*^	This study
pAS216	pCT2 with *6His::frzCD*^*E182D*^	This study
pAS217	pCT2 with *6His::frzCD*^*E168A E175A*^	This study
pAS218	pCT2 with *6His::frzCD*^*E168D E175D*^	This study
pAS221	pCT2 with *6His::frzCD*^*E168A E175A E182A*^	This study
pAS222	pCT2 with *6His::frzCD*^*E168D E175D E182D*^	This study

### Reversal frequency analysis

Cells were grown to mid-log phase and 10 μl of culture were spotted on 1/2 CTT (Hodgkin and Kaiser, 1977) with 1.5% agar. The cells were covered immediately with an oxygen permeable membrane (Yellow Springs Instrument) and allowed to settle for 1 h. Cells were filmed using a Labophot 2 microscope and a micropublisher 3.3 RTV digital camera (Q imaging) and reversal frequencies were analysed by eye. Only moving cells that did not touch another cell during filming were analysed. A students *t*-test (two-tailed, type 3) was used to determine if the reversal frequency of each strain differed from wild type. Strains with a *P*-value less than 0.005 were considered to have different reversal frequencies from wild type. For each strain, a minimum of five movies were taken on two separate days.

### Protein purification from *E. coli*

To obtain soluble protein, *frzF* and *frzF*^*cheR*^ amplified by PCR from genomic DNA and inserted into pET28a (Novagen) at the EcoRI and HindIII sites. The plasmid was sequenced and analysed using the Sequencher program (Gene Codes) and transformed into the *E. coli* Tuner protein expression strain (Novagen). Cells were grown to mid-log phase at 37°C and expression was induced by adding 1 mM of IPTG and transferring cells to 18°C for 16 h. Cells were isolated by centrifugation at 9000 *g*. Cells were lysed by sonication (Branson sonifier 450) (3 × 1 min output 4) in 20 mM Tris pH 7.2, 20 mM imidizole, 10% glycerol, 0.1% CHAPS buffer, 500 mM NaCl lysis buffer supplemented with 2% mammalian protease inhibitor cocktail (Sigma). Insoluble material was removed by centrifugation (20 min, 17 000 *g*) and filtration through a 0.22 micron filter (Corning 115 ml filter system). Cell lysate was injected onto a 5 ml HisTrap HP Nickel column (GE Healthcare) via an AKTA FPLC (GE Healthcare). Protein was washed with 50 ml of lysis buffer, then 50 ml of lysis buffer with 60 mM, 100 mM and 150 mM imidizole. FrzF^CheR^ was eluted using lysis buffer with 250 mM imidizole. The protein was concentrated using 30 000 molecular weight cutoff Centriprep columns (Amicon).

### *In vitro* methylation of FrzCD

Each FrzCD methylation reaction mixture contained 3.5 μM FrzCD, 6 μM SAM, 7 μM FrzF or FrzF^CheR^, 10 mM TrisHCl pH 7.0, 1% glycerol and 50 mM KCl. Reactions took place at 32°C for 4 h. FrzCD methylation was observed initially by SDS-PAGE analysis and then by Mass Spectrometry (see below). Each reaction was repeated in triplicate on four separate days.

### Sample preparation for mass spectrometry

Methylation of receptors was performed as described above. Methylated and unmethylated species were separated by SDS-PAGE. Gels were stained with Coloidal Coomassie (Invitrogen) and bands corresponding to unmethylated and methylated FrzCD were excised and diced into ∼1–2 mm^3^ pieces. The gel slices were destained twice by incubation with 50 μl of 100 mM ammonium bicarbonate and 50 μl of acetonitrile at 37°C for 10 min The gel slices were then dehydrated by incubation with 50 μl of acetonitrile at 37°C for 5 min. FrzCD contains no cysteine residues, so reduction and alkylation were not performed. Gel slices were incubated overnight at 37°C in 50 μl of 100 mM ammonium bicarbonate containing 150 ng of trypsin (Promega), chymotrypsin (Roche) or GluC (Roche). Peptides were extracted from the gel slices by incubation with 30 μl of extraction solution (2% acetonitrile and 1% formic acid) at 37°C for 30 min. The solution was removed and a second extraction was performed with 12 μl of acetonitrile and 12 μl of extraction solution at 37°C for 30 min. The peptide extract was dried down and reconstituted in 25 μl of 0.1% trifluoroacetic acid (TFA).

### Liquid chromatography/mass spectrometry

High-performance liquid chromatography grade water and acetonitrile (Optima) and formic acid (Acros Organics) were purchased from Fisher Scientific. TFA was purchased from Sigma-Aldrich. Peptides were desalted and concentrated on a reversed-phase cartridge (Zorbax C_18_; 5 mm by 0.3 mm i.d.; 5 μm; Agilent) then loaded onto a reverse-phase column (ProteoPep^TM^ C18, 5 cm by 50 μm i.d.; 300 Å; 5 μm; New Objective) using an Ultimate^TM^ nanoliquid chromatography system (Dionex/LC Packings) coupled to a ThermoFinnigan Orbitrap tandem mass spectrometer equipped with a nanospray source (Michrom Bioresources). The column was equilibrated for 5 min in 94% solvent A (0.1% formic acid) and 6% solvent B (90% acetonitrile, 0.1% formic acid). Solvent B was increased linearly to 40% at 40 min, 60% at 45 min and 100% B at 45.1 min, where it was held for 3 min. It was then set back to its initial solvent composition (6% B), where it was held for the duration of the run (60 min). MS survey scans were performed in the orbitrap followed by subsequent MS/MS scans of the three most abundant ions fragmented in the linear ion trap, with a dynamic exclusion of 30 s. Data files (.dta) for MS/MS spectra were generated by Bioworks Browser 3.2 efi software (Thermo Fisher Scientific), converted to MASCOT generic format (.mgf), and searched against a proteomic database for *M. xanthus*. Peptides identified by MASCOT, with or without methyl modifications, at a significance score above 95% confidence, were validated manually. No fixed modifications were used in the searches while methionine oxidation, asparagine and glutamine deamidation, and aspartate and glutamate methylation were used as variable modifications. Three replicates of each sample were analysed.

### Construction of mutants

All strains and plasmids are listed in Table 5. Point mutations were made using PCR. *M. xanthus* genomic DNA from strain DZ2 was used as a template for PCR. Oligonucleotides were prepared by operon. Complementary forward and reverse primers were used to amplify the gene with platinum HiFi Taq (Invitrogen). The primers for the coding strand are listed below with the lower case indicating the altered codon:

For *frzCD*^*E168D*^ catgagacgtcctccacggaccaggcggcggccatccacg, For *frzCD*^*E168A*^ catgagacgtcctccacggcgcaggcggcggccatccacg, For *frzCD*^*E175D*^ caggcggcggccatccacgacacgaccgccaccatggaggag, For *frzCD*^*E175A*^ caggcggcggccatccacgcgacgaccgccaccatggaggag, For *frzCD*^*E182D*^ Cacgagacgaccgccaccatggaggacctgaagcacgcgtcggcgc, For *frzCD*^*E182A*^ CACCATGGAGgcgctgaagcacgc. For *frzCD*^*E168D E175D*^ we used pAS215 as template and primer catgagacgtcctccacggaccaggcggcggccatccacg, For *frzCD*^*E168A E175A*^ we used pAS212 and primer catgagacgtcctccacggcgcaggcggcggccatccacg, For *frzCD*^*E168D E175D E182D*^ we used pAS218 as template and primer cacgagacgaccgccaccatggaggacctgaagcacgcgtcggcgc, For *frzCD*^*E168A E175A E182A*^ we used pAS217 (E168A E174A) and primer CACCATGGAGgcgCTGAAGCACGC.

Once each half of the insert was constructed the two PCR products per mutant were used as template and the primers. GATATCCAGCTGCCCGAGGAGGACGATG and GGCCAGTGCCAAGCTTCATTACTAGTCG were used to construct the complete insert. The insert was then placed into the digested (SmaI and HindIII) pCT2 (Mignot *et al*., 2007) plasmid via the In-Fusion reaction (Clonetech). The resulting plasmids were confirmed by DNA sequencing.

Plasmids containing the wild-type *frzCD* and the point mutations were inserted into the *crtB* locus of *M. xanthus* strain Δ*frzCD* and confirmed by PCR and sequencing. In all figures ‘wild type’ corresponds to *6His::frzCD* in the *crtB* locus. To induce expression of *frzCD* and the point mutants*,* 50 μg ml^−1^ of anhydrotetracycline HCl (Reidel-de Haen) was used in all media.

### Immunoblot analysis of FrzCD

*Myxococcus xanthus* strains were grown to mid-exponential phase, concentrated by centrifugation, and resuspended in 1 × SDS loading buffer lacking coloured dye. Cells were lysed by 10 s of sonication (Branson sonifier 450) on ice. Protein concentration was determined using the BCA (bicinchoninic acid) method (reagents from Pierce). Cells were resuspended to the same concentration in 2 × SDS loading buffer. Thirty micrograms of total protein were loaded per lane on 10% Tris HCl ready gels (Bio-Rad). After electrophoresis, the gel was transferred to a nitrocellulose membrane. Blots were probed with anti-FrzCD antibody as described (McCleary *et al*., 1990) and with the antirabbit alexa fluor 680 (Molecular Probes) secondary antibody. Blots were visualized using an infrared imaging system (LiCor) and results were analysed using Odyssey software. The data were confirmed by three independent experiments done in duplicate.

## Supplementary material

This material is available as part of the online article from: http://www.blackwell-synergy.com/doi/abs/10.1111/

j.1365-2958.2008.06323.x

(This link will take you to the article abstract).
