# Supplementary material for: Site-specific receptor methylation of FrzCD in Myxococcus xanthus is controlled by a tetra-trico peptide repeat (TPR) containing regulatory domain of the FrzF methyltransferase
Source: Mol Microbiol. 2008 Jun 19;69(3):724–35. doi: 10.1111/j.1365-2958.2008.06323.x (PMC2535941; doi:10.1111/j.1365-2958.2008.06323.x)
Supplement: Supplementary file 1 [file mmi0069-0724-SD1.pdf]

Supplemental Material

Table S1. Ions from a MS/MS of a chymotryptic fragment[1] of FrzCD show that FrzCD is not methylated

AA[2]	Theoretical[5] b-ions[3]	Observed[6] b-ions	Theoretical y-ions[4]	Observed y-ions
A171	1500.6	1500.4		
A172			1361.6	1361.3
I173	842.9 (2+)	842.7 (2+)		
H174				
E175				
T176	1026.5 (2+)	1026.3 (2+)		
T177	1077.0 (2+)	1077.0 (2+)	810.4	810.1
A178	1112.5 (2+)	1112.5(2+)	709.3	709.1
T179	1163.0 (2+)	1163.0 (2+)	638.3	638.1
M180	1236.6 (2+)	1236.3 (2+)	537.2	537.1
E181	1301.1 (2+)	1300.8 (2+)		

[1]No ions were observed for residues $_{157}$AASTQHETSSTEQA$_{170}$ of the chymotryptic peptide $_{157}$AASTQHETSSTEQAAAIHETTATMEEL$_{183}$ (Fig. S4), so these residues were removed from the left column for simplicity.

[2]Amino acids are indicated by their one letter code; numbers represent their position in FrzCD. The # symbol represents an amino acid that is methylated.

[3]A b-ion is an amino-terminal charged fragment generated after ion activation causes a peptide bond to break

[4]A y-ion is a carboxy-terminal charged fragment generated after ion activation causes a peptide

bond to break

[5] Theoretical ions are calculated by dividing the predicted mass of an ion by the ion's charge ($M/Z = (M+n*H^+)/n$).

[6] Observed ions were found by digesting FrzCD with chymotrypsin and using tandem MS-MS.

[7] All ions are 1+ charged unless otherwise indicated in parentheses

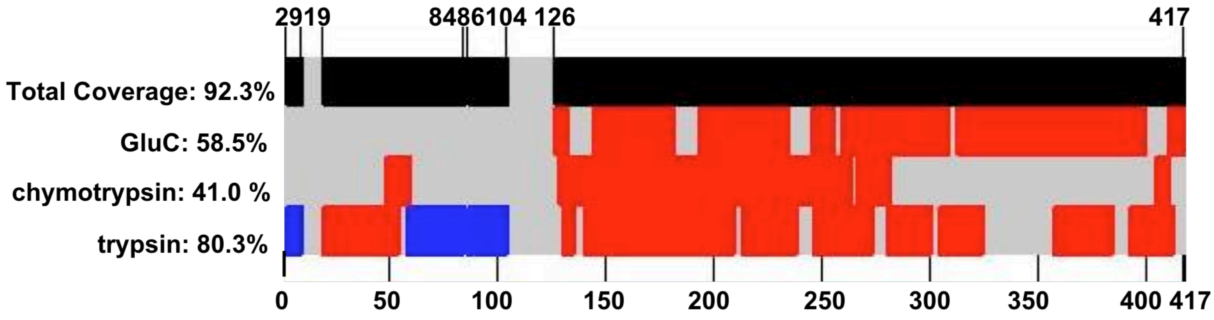

Figure S1. Three proteases provide 92.3% FrzCD sequence coverage. The top row displays total FrzCD sequence coverage obtained by mass spectrometry from all three enzymes. The second, third, and fourth row show FrzCD sequence coverage for GluC, chymotrypsin, and trypsin, respectively. Black indicates total protein coverage, grey indicates lack of coverage, red indicates protein coverage obtained through tandem mass spectrometry, blue indicates protein coverage obtained through mass mapping. Numbers indicate the amino acid position in FrzCD.

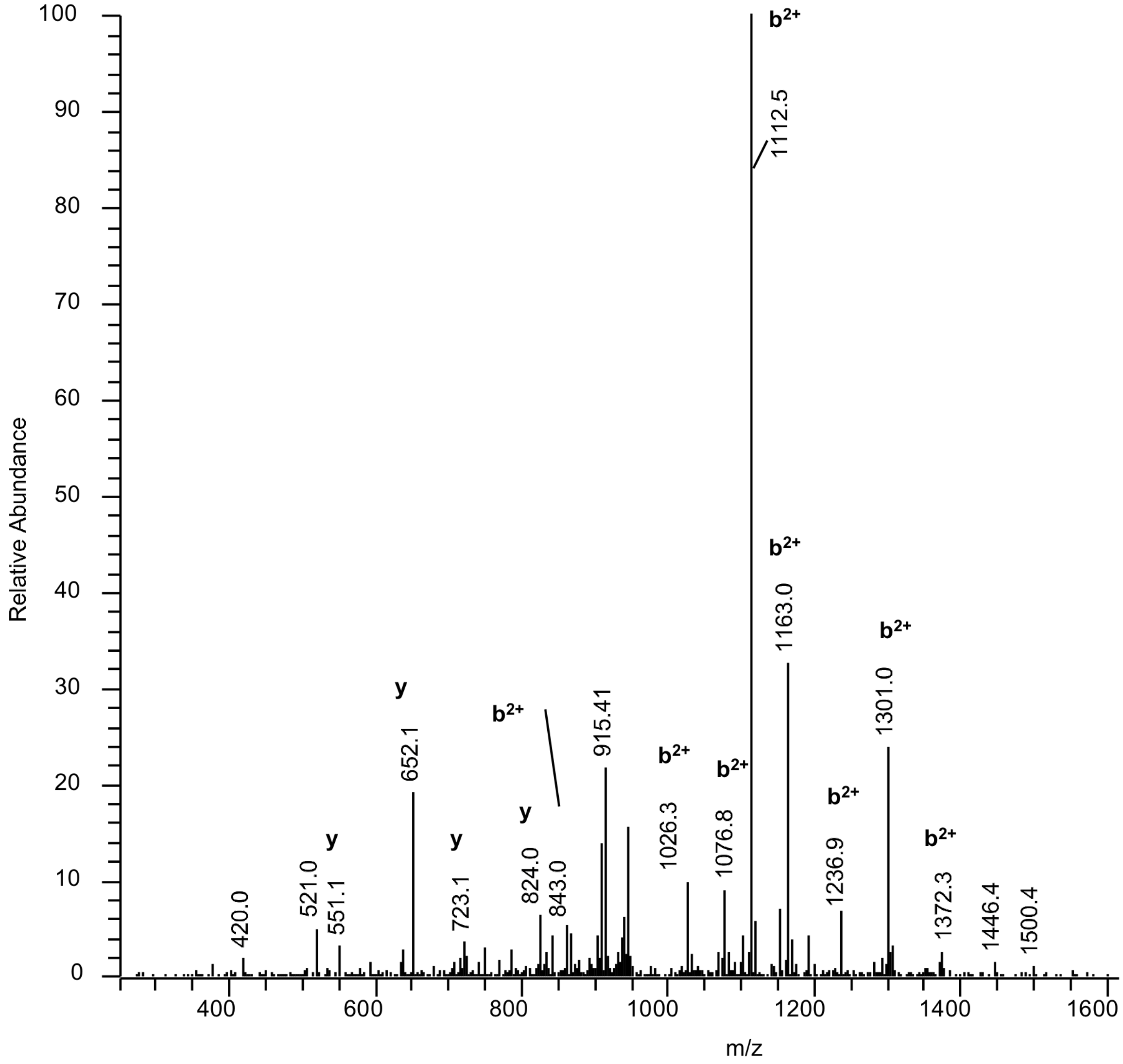

Figure S2. MS spectrum for peptide $_{157}$AASTQHETSSTEQAAAIHETTATMEEL$_{183}$ of FrzCD. y- and b-ions confirm that FrzCD is methylated on residue E182 by FrzF. See table 1 for predicted y- and b-ions. The peptide was generated using chymotrypsin.

A.

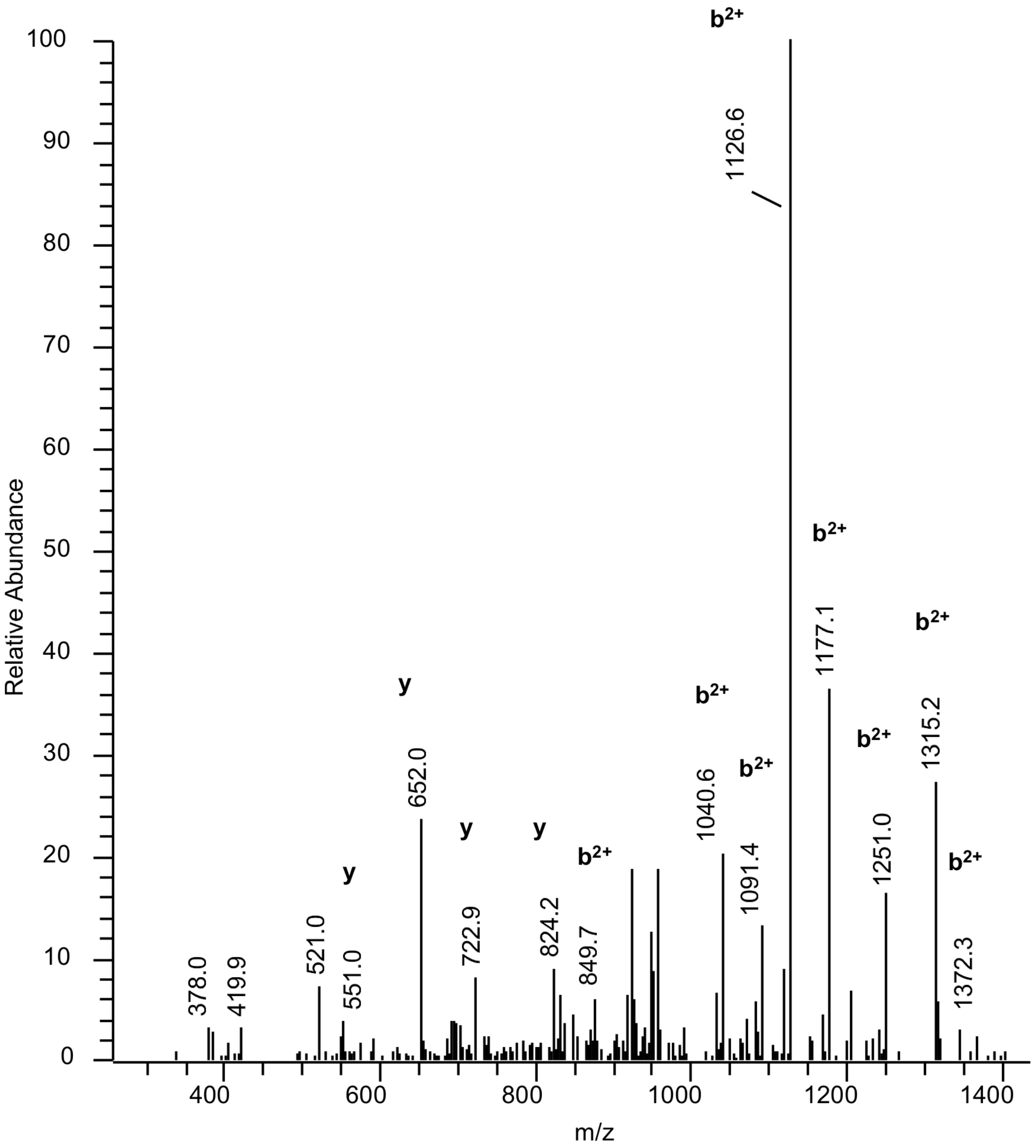

Relative Abundance
m/z
100
90
80
70
60
50
40
30
20
10
0
400
600
800
1000
1200
1400
b2+
1126.6
b2+
1177.1
b2+
1315.2
y
652.0
b2+
1040.6
b2+
1091.4
b2+
1251.0
y
722.9
y
824.2
b2+
849.7
y
551.0
b2+
1372.3
378.0
419.9
521.0

B.

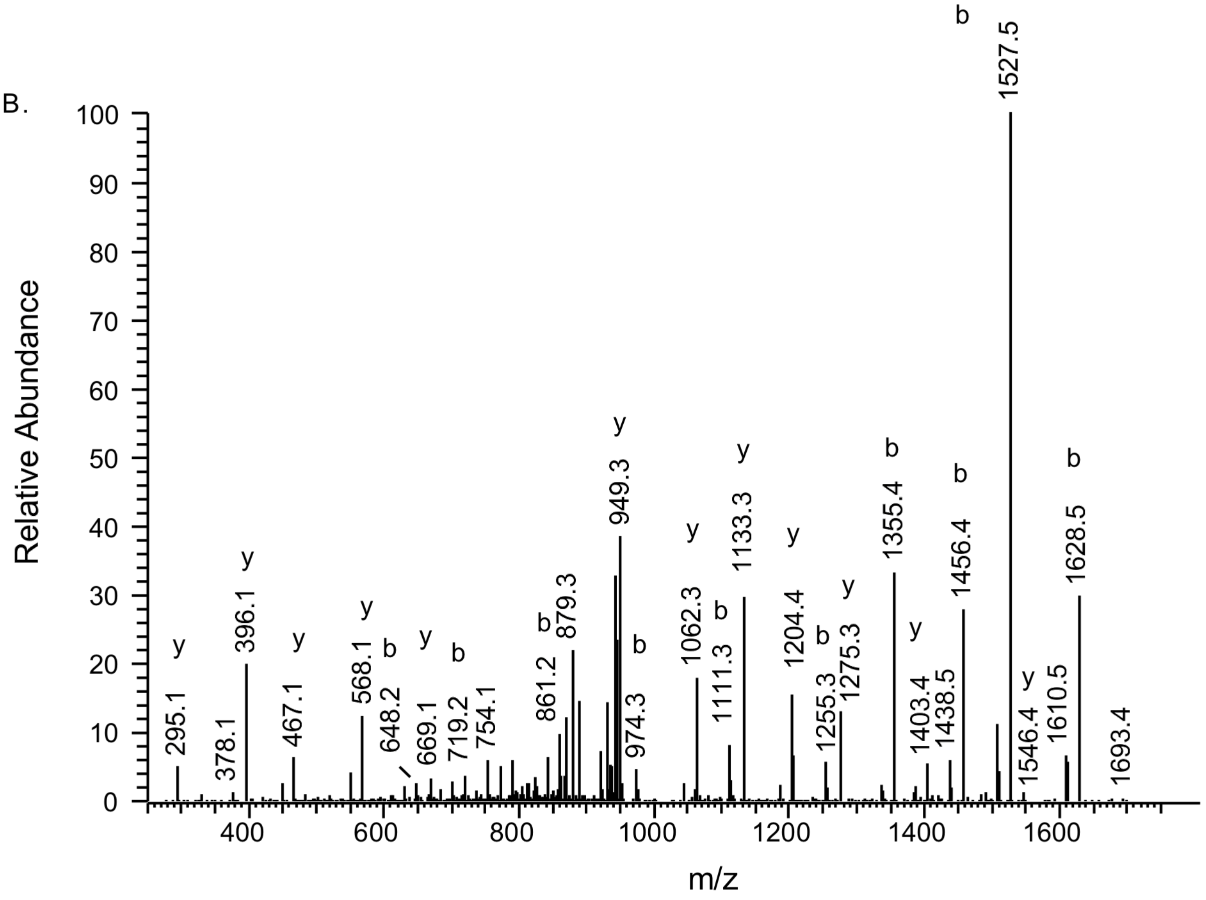

Figure S3. A. MS spectrum for peptide $_{157}$AASTQHETSSTEQAAAIHETTATMEL$_{183}$ of FrzCD. y- and b-ions confirm that FrzCD is methylated on residues E175 and E182 by FrzF^{CheR}. See table 2 for predicted y- and b-ions. The peptide was generated using chymotrypsin. **B.** MS spectrum for peptide $_{164}$TSSTEQAAAIHETTATME$_{181}$ of FrzCD. y- and b-ions confirm that FrzCD is methylated on residues E168 and E175 by FrzF^{CheR}. See table 3 for predicted y- and b-ions. The peptide was generated using GluC.

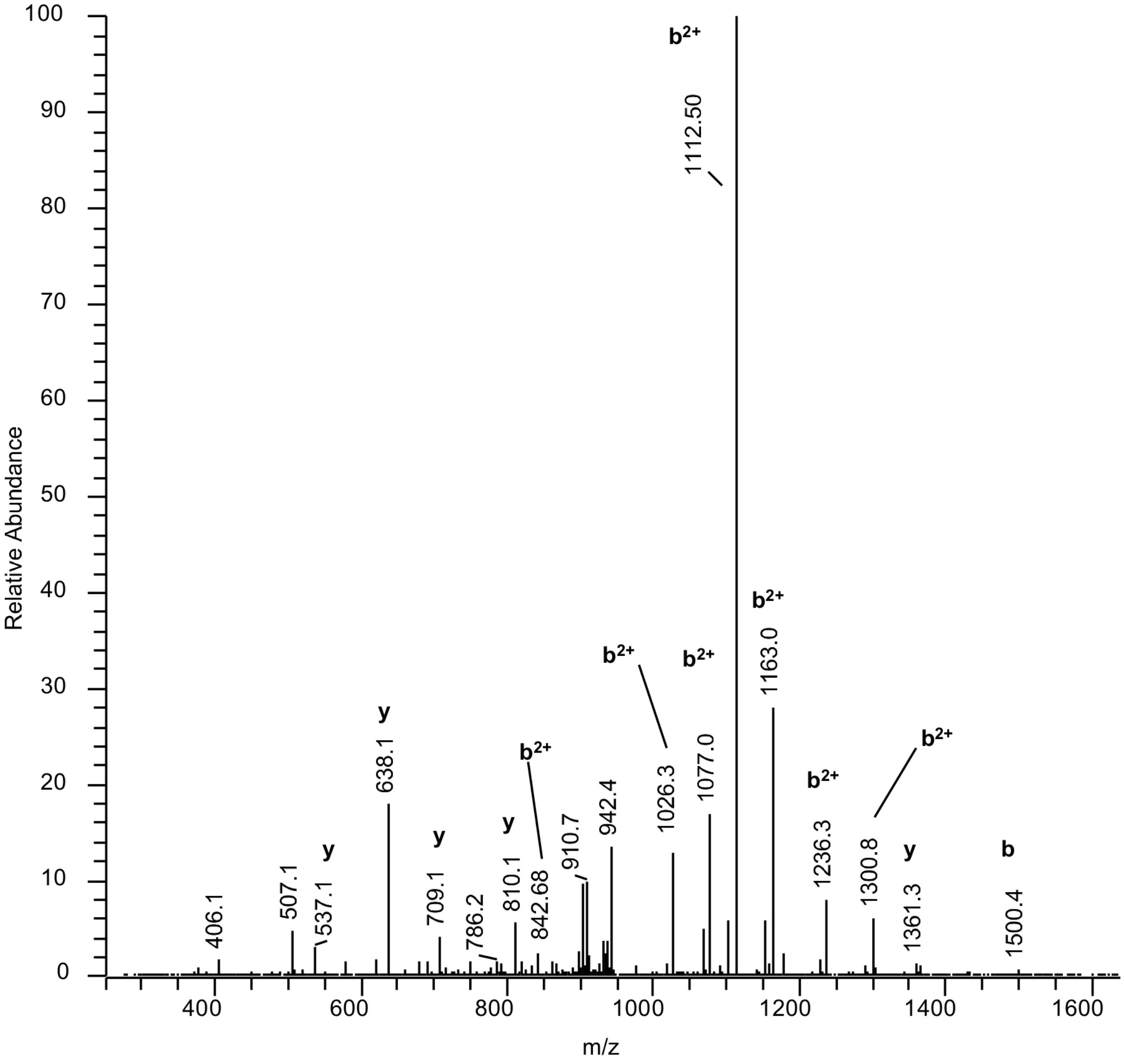

Figure S4. MS spectrum for peptide $_{157}$AASTQHETSSTEQAAAIHETTATMEL$_{183}$ of FrzCD. The Y-axis represents the relative abundance of each ion and the X-axis represents the mass/charge ratio of each ion. y- and b-ions confirm that FrzCD is not methylated in the absence of FrzF. See table S1 for predicted y- and b-ions. The peptide was generated using chymotrypsin.

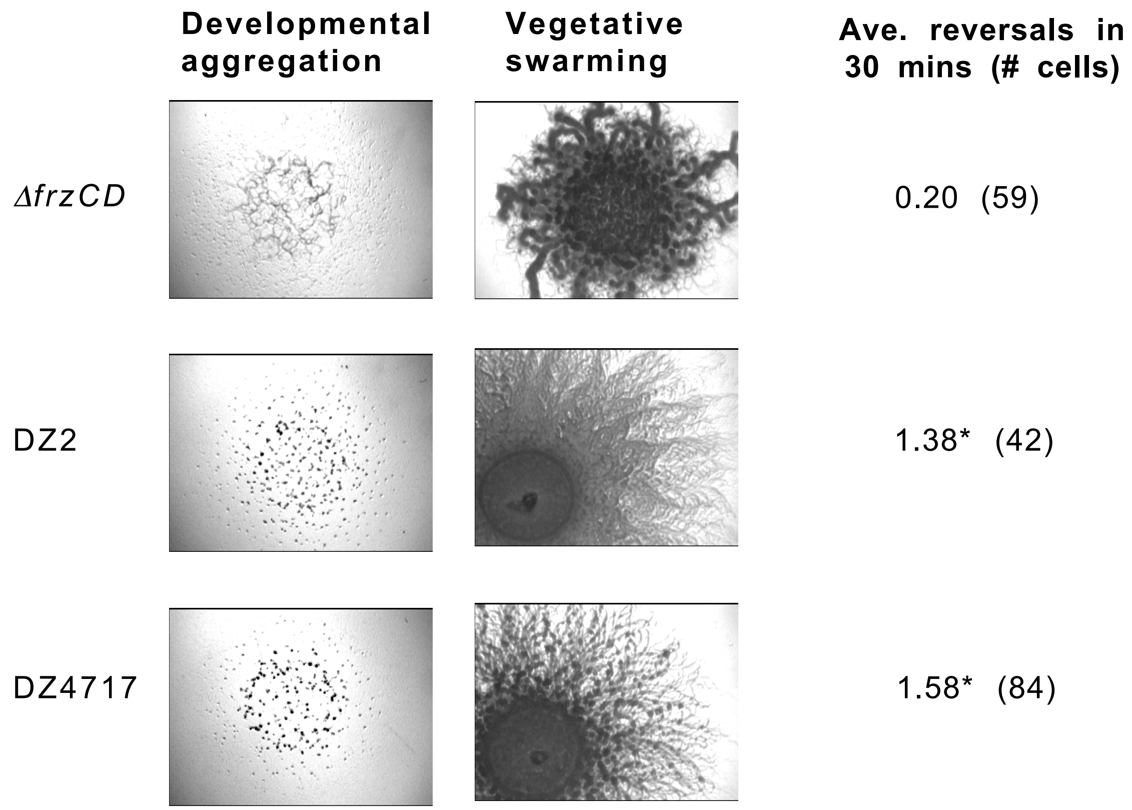

Figure S5. Phenotypes of *ΔfrzCD*, DZ2 (wild type) and DZ4717 (*6His::frzCD*). The left panel shows aggregates/fruiting body formation after four days on starvation media containing hard agar. The middle panel shows the ability of groups of cells to swarm on rich media containing soft agar. The right panel shows the average number of individual cell reversals on hard agar in 30 minutes. The number of cells analyzed per strain is indicated in parenthesis. Asterisks (*) indicate values that are not statistically different from each other based on a student's T-test.
